# ‘Everybody knows’, but the rest of the world: the case of a caterpillar-borne reproductive loss syndrome in dromedary camels observed by Sahrawi pastoralists of Western Sahara

**DOI:** 10.1186/1746-4269-9-5

**Published:** 2013-01-10

**Authors:** Gabriele Volpato, Antonello Di Nardo, Davide Rossi, Saleh M Lamin Saleh, Alessandro Broglia

**Affiliations:** 1 Department of Social Sciences, Wageningen University, Wageningen, The Netherlands; 2 Institute of Biodiversity, Animal Health and Comparative Medicine, University of Glasgow, Glasgow, UK; 3 The Pirbright Institute, Pirbright, Woking, Surrey, UK; 4 Faculty of Veterinary Medicine, University of Bologna, Bologna, Italy; 5 Sahrawi Veterinary Services, Ministry of Public Health, Sahrawi Arab Democratic Republic, Rabouni, Algeria; 6 SIVtro Vétérinaires Sans Frontières, Italy, Legnaro, (PD), Italy; 7 Africa ‘70 (International Non-Governmental Organization), Monza, (MI), Italy; 8 Scientific Panel on Biological Hazards, European Food Safety Authority, Parma, Italy

**Keywords:** *Lasiocampidae*, Abortion, Neonatal loss, Dromedary camel, Reproductive loss syndrome, *Duda*, MRLS, Ethnoveterinary medicine, Community-based participatory research

## Abstract

**Background:**

The traditional knowledge of local communities throughout the world is a valuable source of novel ideas and information to science. In this study, the ethnoveterinary knowledge of Sahrawi pastoralists of Western Sahara has been used in order to put forward a scientific hypothesis regarding the competitive interactions between camels and caterpillars in the Sahara ecosystem.

**Methods:**

Between 2005 and 2009, 44 semi-structured interviews were conducted with Sahrawi pastoralists in the territories administered by the Sahrawi Arab Democratic Republic, Western Sahara, using a snow-ball sampling design.

**Results:**

Sahrawi pastoralists reported the existence of a caterpillar-borne reproductive loss syndrome, known locally as *duda*, affecting their camels. On the basis of Sahrawi knowledge about *duda* and of a thorough literature review, we built the hypothesis that: 1) caterpillars of the family Lasiocampidae (genera *Lasiocampa*, *Psilogaster*, or *Streblote*) have sudden and rare outbreaks on *Acacia* treetops in the Western Sahara ecosystem after heavy rainfall; 2) during these outbreaks, camels ingest the caterpillars while browsing; 3) as a consequence of this ingestion, pregnant camels have sudden abortions or give birth to weaklings. This hypothesis was supported by inductive reasoning built on circumstantiated evidence and analogical reasoning with similar syndromes reported in mares in the United States and Australia.

**Conclusions:**

The possible existence of a caterpillar-borne reproductive loss syndrome among camels has been reported for the first time, suggesting that such syndromes might be more widespread than what is currently known. Further research is warranted to validate the reported hypothesis. Finally, the importance of studying folk livestock diseases is reinforced in light of its usefulness in revealing as yet unknown biological phenomena that would deserve further investigation.

**Resumen:**

‘*Todos lo saben*’, *menos el resto del mundo*: *el caso de un síndrome de pérdida reproductiva en dromedarios transmitido por orugas y observado por pastores nómadas saharauis del Sáhara Occidental*.

**Antecedentes:**

Los conocimientos tradicionales de las comunidades locales de todo el mundo son una valiosa fuente de nuevas ideas e información para la ciencia. En este estudio, se utilizaron los conocimientos de etnoveterinaria de pastores saharauis del Sáhara Occidental con el fin de proponer una hipótesis científica sobre las interacciones competitivas entre los camellos y las orugas en el ecosistema del Sáhara.

**Métodos:**

Entre los años 2005 y 2009, se realizaron 44 entrevistas semi-estructuradas a los pastores saharauis en los territorios administrados por la República Árabe Saharaui Democrática, Sáhara Occidental, mediante un diseño de muestreo por bola de nieve.

**Resultados:**

Los pastores nómadas saharauis describieron un síndrome reproductivo transmitido por orugas, llamado *duda*, entre sus camellas. Sobre la base de los conocimientos saharauis sobre el *duda* y una revisión literaria exhaustiva, se propuso la siguiente hipótesis: 1) brotes esporádico de orugas de la familia Lasiocampidae (géneros *Lasiocampa*, *Psilogaster* o *Streblote*) en árboles de *Acacia* se pueden presentar después de fuertes lluvias en el ecosistema del Sáhara Occidental; 2) durante estos brotes, los camellos ingieren las orugas durante el pastoreo; 3) como consecuencia de esta ingestión, se producen abortos repentinos o partos de crías debilitadas. Apoyamos esta hipótesis mediante razonamiento inductivo basado en evidencia circunstancial y razonamiento analógico con síndromes similares en yeguas de los Estados Unidos y Australia.

**Conclusiones:**

Este es el primer reporte de la posible existencia de un síndrome de pérdida reproductiva en camellos, transmitido por orugas. Se insinúa que estos síndromes son más comunes que lo que actualmente se conoce. Se sugieren investigaciones adicionales para poner a prueba nuestra hipótesis. Finalmente, se destaca la importancia de estudios de las enfermedades del ganado en pueblos de pastores nómadas porque pueden revelar fenómenos biológicos aún desconocidos y merecen ser investigados.

## Introduction

In the scientific arena, discoveries are based on generating hypotheses, collecting data, and examining whether a correlation exists, thus testing its validity. In any case, discoveries are based on a novel idea to be tested, and these novel ideas are not always straightforward. They ought to be based on previous knowledge about the phenomenon to be investigated, or on analogies drawn by similar phenomena. In field research, novel ideas on biological and ecological phenomena may be found in the traditional knowledge of local communities throughout the world. The knowledge accumulated by these populations has already contributed to several aspects of the scientific research (e.g. health and nutrition or conservation and management of natural resources and ecosystems) [1]. In this paper, knowledge about camel illnesses drawn from Sahrawi pastoralists of Western Sahara will be used to evaluate the potential interactions between dromedary camels (*Camelus dromedarius* L.) and caterpillars in the Sahara ecosystem. The novel idea on which this hypothesis is based is the observation by Sahrawi pastoralists of camels’ behaviour and health (i.e. that the ingestion of *Lasiocampidae* caterpillars causes a reproductive loss syndrome), and is thus part of Sahrawi ethnoveterinary knowledge.

Ethnoveterinary knowledge is of great importance for its role among pastoralists across the world, and its study and application can be of valuable interest to Western veterinary medicine [2]. In recent decades, several studies have given attention to ethnoveterinary knowledge [3,4], although their focus has been directed towards local remedies and practices and their validation rather than to the local conceptualization of livestock illnesses and their investigation [5-8]. This is especially true for camels. There is a lack of information on camel diseases (e.g. on aetiological factors, epidemiological patterns, symptoms, prevention and treatments) [9], particularly concerning nomadic management systems. New and little known diseases have been reported in recent years [9,10], and this has been achieved on the basis of the knowledge and observations of local communities. The exact causes of several camel diseases still remain unknown, and pastoralists’ lore ‘*offers intriguing clues to modern veterinarians who are trying to establish and characterise the aetiology of the diseases and ultimately find effective treatments*’ [10]. Pastoralists’ ethnoveterinary knowledge, thus, can help in the identification of previously unrecognised diseases, which need to be studied and understood in order to develop appropriate management strategies and treatments.

In this paper, a camel reproductive loss syndrome – namely *duda* syndrome and whose cause is allegedly the ingestion by pregnant camels of caterpillars living in *Acacia* treetops – observed by Sahrawi pastoralists of Western Sahara is reported. The logical reasoning process is based on: 1) the description of *duda* syndrome in Western Sahara as understood by Sahrawi pastoralists; 2) the likelihood for this syndrome to explain reality on the basis of available information about camel diseases and the Western Sahara ecosystem (i.e. rain patterns, moth species and their reproductive cycles); 3) the analogical link with other similar syndromes described in ruminant species. The hypothesis of the existence of a poorly known competitive interaction between camels and *Lasiocampidae* caterpillars in the Sahara ecosystem will be discussed, emphasizing the usefulness of a thorough evaluation of folk livestock diseases among pastoralists in the field of ethnoveterinary studies.

## Background

*Sahrawi*, literally ‘people from the desert’, is the name given to nomadic tribes who traditionally inhabited the coastal area of north-western Africa, which includes Western Sahara, Northern Mauritania, and part of south-western Algeria. Sahrawi people were essentially nomadic, raising camels, goats, and sheep in the rocky and sandy low-lying plains of the above defined area and relying for food on camel milk and meat, dates, sugar, cereals and legumes bartered for livestock in local markets [11-13]. In 1975, fifty years of Spanish colonial rule ended and following the occupation of Western Sahara by Morocco, about 70,000 Sahrawi became refugees after fleeing the Moroccan army [14]. Nowadays, about 165,000 Sahrawi live in four refugee camps located on a desert plateau called *Hamada*, near the Algerian city of Tindouf [15].

Besides the camps, Sahrawi – through their political representative, the *Polisario Front* – also control the eastern part of the Western Sahara, which was taken away from Moroccan control through a guerrilla war that lasted until the peace agreement of 1991 [16]. These inland areas of Western Sahara are the so-called ‘liberated territories’ (approximately 20% of the Western Sahara), while the remaining is under the administering authority of the Moroccan government. About 20–30,000 Sahrawi nomads inhabit the liberated territories, crossing them with their herds and using refugee camps, Tindouf, and Zouérat in Mauritania as main commercial hubs.

Traditionally, the camel was central to Sahrawi livelihood and culture (e.g. myths, symbolic representations, beliefs, cultural identity, etc.). Camels were used for food, leather, wool, and to obtain medicinal and veterinary remedies. Fresh camel milk was the basis of Sahrawi diet, and meat and fat were also important. Camels served also as the main means of transport of both humans and goods throughout the desert [13]. Their ecological adaptations, coupled with cultural and behavioural aspects of nomadic life, allowed Sahrawi tribes to exploit the biologically-poor territory of Western Sahara for centuries and to serve as a commercial and cultural bridge between Sub-Saharan and North-Saharan populations. Nowadays, camel husbandry is still practiced in the liberated territories by Sahrawi nomads and, to a lesser extent, by refugees in the surroundings of the camps [17].

In order to be successful in their herd management in the arid ecosystem, the Sahrawi developed profound knowledge of local ecology, camel ethology, and veterinary medicine. The reproductive loss syndrome discussed in this paper is part of this knowledge that the Sahrawi have built, accumulated, and passed on between generations in order to maintain healthy herds and thus reproduce their system of subsistence.

### Study area and its climate

The area under study includes the ‘liberated territories’ of Western Sahara, northern Mauritania, and the part of Algeria to the south and south-east of the *Hamada* of Tindouf, which are the customary nomadic territories of Sahrawi pastoralists. Across this area, the climate is continental: summer daytime temperatures pass 50°C, while winter night time temperatures may drop to 0°C. Rainfalls are torrential, unpredictable, and patchy, with an average annual rainfall of 30-50 mm. Generally occurring from the end of the summer through autumn, these rains are driven by the extreme northerly penetration of the African Monsoon from the south, or are associated with the Atlantic Westerlies [18]. Rains are highly irregular within and between years, and there are recurrent droughts.

Biogeographically, we can distinguish two main areas: *Zemmur* to the North, and *Tiris* to the South. The first runs east–west between northern Western Sahara and northern Mauritania: it is characterised by sand and gravel plains with occasional surface of sandstone and granite in its eastern and central parts, and by higher relief and hilly terrain in its western part. All *Zemmur*, and especially its central and western areas, is drained by inactive or occasionally active river channels that flow west into the *Saguia elHamra*, a large ephemeral river. After the rains, *Zemmur* displays a savannah-like environment dominated by Acacia-Panicum vegetation, while flowering prairies may appear on flat gravel areas. The southern sector, known as *Tiris*, is more arid and characterized by flat sand and gravel plains from which characteristic black granite hills arise in either clusters or in isolation. In *Tiris*, there are no dry riverbeds, and hence vegetation is mostly herbaceous, adventicious, and includes large areas covered by halophytic plants [19].

## Methodology

The data analysed in this paper were collected in the Sahrawi refugee camps and in the Polisario-controlled Western Sahara between 2005 and 2009. Investigation methods included semi-structured interviews [20] with Sahrawi pastoralists and camel herders. Informants were identified through a snow-ball sampling design and by approaching nomads’ tents in the ‘liberated territories’. Semi-structured interviews dealt with data collection of the aetiology, epidemiology, symptoms, treatment, and prevention of the reported syndrome, including aspects of caterpillars’ ecology, camels’ feeding and rain patterns. Furthermore, the interviews aimed at recalling episodes of reproductive loss syndrome in pastoralists’ herds. A total of 44 semi-structured interviews were carried out.

Interviews were conducted in *Hassaniya* (the Arabic language with Berber substrate spoken by the Sahrawi), recorded and translated into Spanish by local research assistants. In every case, prior informed consent was obtained verbally before the interview was conducted, according to the ethical guidelines adopted by the American Anthropological Association [21] and by the International Society of Ethnobiology [22].

## Results and discussion

### General description of the syndrome by Sahrawi pastoralists

**Figure 1:**
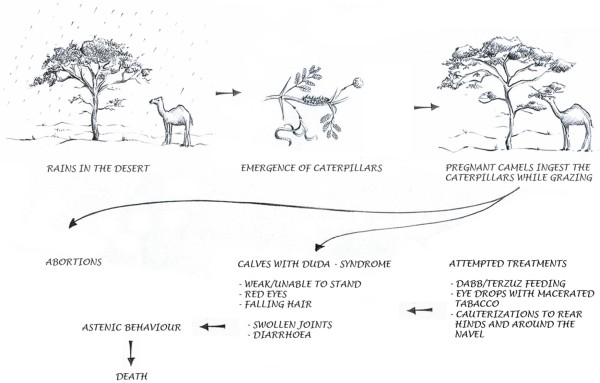
**Schematic representation of the *duda *syndrome reconstructed from field interviews with Sahrawi pastoralists(Author:Pavlína Kourková).**

Among the numerous camel illnesses recognized by the Sahrawi, they describe a camel reproductive loss syndrome called *duda*. *Duda* is the generic Arabic name for ‘worm’ and ‘caterpillar’. The story, as told by Sahrawi, goes like this: when a pregnant camel ingests, while browsing, the caterpillars that live on *Acacia* trees, it ‘transmits’ them to its foetus, and this may cause abortions or the delivery of a weak and premature calf. Ingestion of *duda* is reported to be of no harm to adult animals. A schematic representation of the syndrome is shown in Figure 1. Informants stated that ‘*you never find the duda in the calf*’, and nonetheless they bore no doubts that the ingestion of these caterpillars is the cause of the syndrome. Sahrawi described it as a highly seasonal and rare syndrome, as the caterpillars considered to be responsible proliferate on *Acacia* trees only after rains and only occasionally their population outbreaks. When this happens, an abortion storm [23] is likely to occur.

#### Symptomatology

The clinical signs of *duda* in pregnant camels and in calves described by Sahrawi pastoralists are provided in Table 1. Abortions are reported by all the informants and hence are the key sign of *duda*. Sahrawi pastoralists stated that, after heavy rains and caterpillar outbursts, *duda* may cause a high rate of abortion, and that most abortions occur suddenly to camels at a mid-late stage of pregnancy. A uterine prolapse may follow. In the scientific literature, abortions in camels are usually associated with idiopathic death of foetus, salmonellosis, trypanosomiasis, campylobacteriosis, trichomoniasis, stress conditions, and poisonous plants, with high incidence of abortion being a major factor in limiting herd size growth and productivity [24,25]. Middle-late term and late-term abortions and premature deliveries are common reproductive disorders of trypanosomiasis-affected camels [26,27]. However, Sahrawi distinguished *duda*-related abortions from other causes of abortion based on their sudden and stormy pattern and on the presence, during or immediately prior to the abortion storm, of caterpillars on *Acacia* foliage.

**Table 1:** **Clinical signs of *duda *as reported by Sahrawi pastoralists during the field interviews**

	**Clinical signs**^†^	**Number of reports (%)**
*Pregnant camels*	Abortion	44 (100)
	Uterine prolapse	7 (16)
*Calves*	Weakness	27 (84.4)
	Red eyes	26 (81)
	Falling hair	26 (81)
	Astasis and incoordination	21 (65.6)
	Joint effusions	12 (37.5)
	Swelling of lymph nodes	8 (25)
	Diarrhoea	7 (22)

^†^N = 44 for abortions, and N = 32 for calf symptoms (twelve informants listed only abortions and uterine prolapse without reference to the *duda* syndrome in calves).

Abortions were considered the main result of *duda* ingestion, with the specification that ‘*if they* [pregnant camels] *do not abort*, *then the newborn dies soon after birth*; *if it does not die*, *it usually grows slowly*, *weak and with problems at the joints*'. Calves that are born with *duda* syndrome present red eyes, falling hair, and a general asthenic behaviour that includes weakness, astasis, ataxia, and sometimes joint effusions. Diarrhoea and swelling of lymph nodes may follow. On the basis of interview reports, three forms can be recognised: 1) Primary signs of the syndrome present frequently at birth, which are weakness, astasis, red eyes, and falling hair; 2) Secondary signs also present at birth but reported by a more limited group of informants, i.e. joint effusions; 3) Secondary signs emerging after birth (a few days to a week), i.e. diarrhoea and swollen head. The ‘fainting’ of the newborn calf (‘*calves with duda fall*, *you lift them up and they fall*’) and red eyes (and ‘*watching upward*’) are regarded by the Sahrawi as clear symptoms of *duda* intoxication. Calves may survive, but more commonly they die after a few days, following refusing to suckle, ‘*isolating themselves*’, and diarrhoea.

To Sahrawi herders, *duda* is the best known example of illnesses that ‘*are born with the calf*’ (i.e. not caused by any external agent after birth). The primary symptoms are consistent with the health condition of premature newborn calves, which are often weak, may have a silky coat, floppy ears, and display an extreme laxity of the joint [28]. Some secondary symptoms (e.g. diarrhoea, joint effusions) are consistent with bacterial infections and sepsis, which are further aggravated by the inability of premature calves to suckle and feed as needed. Also, in light of the fact that in camels there is no antibody transfer from the mother during foetal development, the new-born calf has no natural protection against diseases before drinking the first colostrum. With regard to joint effusions, a condition that affects newborn calves is known: neonatal septic arthritis (or joint ill) causes a swelling of carpal and tarsal joints, and is thought to be due to poor hygiene at parturition and bacterial contamination of the umbilical cord [29].

#### Prevention and treatment

When asked if there is some way of preventing the syndrome, Sahrawi herders stated that this is not possible, due to the fact that caterpillars might outbreak suddenly on *Acacia* trees and that there is no way to prevent camels from ingesting them while grazing. Allegedly, ‘*they swallow these caterpillars without realizing it because they have the same taste as pastures*'. Besides, there is not an effective treatment for calves born with the syndrome. Herders sometimes attempt treatments targeting the symptoms: for calves that are weak and unable to stand, a spiny-tailed lizard (*Uromastyx acanthinura* Bell, Agamidae) – called *dabb* – is cooked, minced, mixed with oil and drenched to the calf. Furthermore, spikes of *terzuz* (*Cynomorium coccineum* L., Balanophoraceae) are fried in oil and fed to the calf. Both *terzuz* and *dabb* are symbols of male power and fertility, and their drenching to the weak calf is thus to be considered as an attempt to ‘give strength’ and ‘ability to stand’ to the animal. Another tentative treatment is to feed the calf with some of its own placenta soon after birth. To treat red eyes, tobacco leaves are macerated in water and applied as eye drops, and in cases of swollen head, macerated tobacco leaves are drenched to the calf, or ‘*tobacco is smoked on his nob*.’ In addition, Sahrawi herders often perform cauterizations with parallel or cross shaped lines on both hindquarters, at the base of the tail, and around the navel when calves are unable to stand and have swollen joints. Admittedly, all these therapies have very limited efficacy, with progress being dependent on the severity of calves’ condition at birth and often ending with the death of the animal.

### Knowledge of duda syndrome amongst other Saharan nomads

Knowledge related to *duda* syndrome is widely diffused and consistent among Sahrawi herders, who generally referred to it as something that ‘everybody knows’. Furthermore, it is not limited to the Sahrawi, but also reported by Mauritanian Moors and by the Tuareg of Niger. Given the little information available on the subject, full descriptions of the syndrome, as reported in other studies, are given below.

A caterpillar-borne syndrome of camel called *asolof* is described by the Tuareg of Niger [30]. Antoine-Moussiaux et al. [30] wrote: ‘Asolof is an urticant caterpillar that lives in the acacias (*A*. *raddiana*) during the rainy seasons. Its ingestion is responsible for abortions among camels. The expulsion of the foetus is often followed by uterine prolapse. Herders blame a reaction ‘of irritation’ of the uterus similar to the one suffered by the skin at contact with the urticant hairs, and that provokes a premature expulsion of the foetus and the observed prolapse'. The authors eventually call for further studies of *asolof*. Notably, the limited historical cultural exchange between Sahrawi and Tuareg and the use of different names (*duda* in Hassaniya and *asolof* in Tamacheq) for the syndrome suggest that the two populations elaborated their observations independently.

The *duda* (or *douda*) syndrome is recognized also by Mauritanian camel herders [31]: among the diseases reported to affect camel herds by veterinary professionals in Mauritania, *duda* was cited by 43% of them [32]. Among Moor pastoralists of Mauritania, the ingestion of caterpillars is not only associated with abortions but also with calf diarrhoeas, and calves affected by *duda* are regarded as particularly predisposed to the latter [33]. In listing the causes of abortions in Mauritanian camels, Diagana [34] refers to ‘camel abortions that would be caused, at the time of tree browsing, notably of TALH (*A*. *radiana*) [sic], to the ingestion of cocoons or caterpillars of lepidopters.’ These abortions are said ‘to be announced by sadness, inappetence, difficult walking, and colic’ [34].

**Figure 2:**
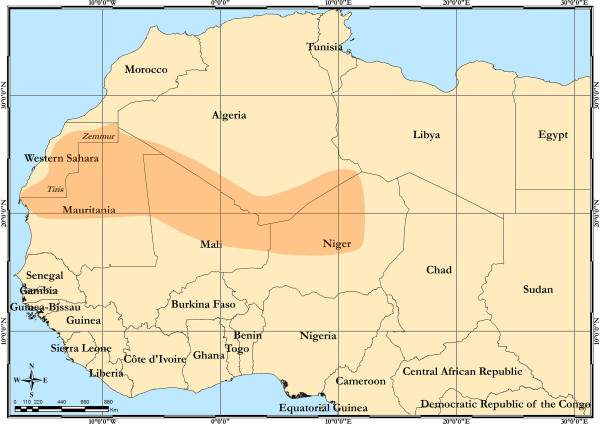
**Distribution of the *duda *syndrome in Africa.**

In spite of an extensive literature search, no other information about caterpillar-borne reproductive loss syndromes in camels was found. In Figure 2, the known cultural distribution of the *duda* syndrome in Africa is depicted. The syndrome seems to be common knowledge amongst camel nomads of the Central and Western Sahara desert, but completely unknown to the rest of the world. These facts beg answers to the following questions: Is *duda* syndrome for real? Is there a direct correlation between caterpillar ingestion and abortions? What species are these caterpillars? Do their numbers outbreak in *Acacia* treetops in favourable conditions? With the limited available knowledge, those questions will be addressed below, and some hypotheses for further research will be proposed.

### The caterpillars

**Figure 3:**
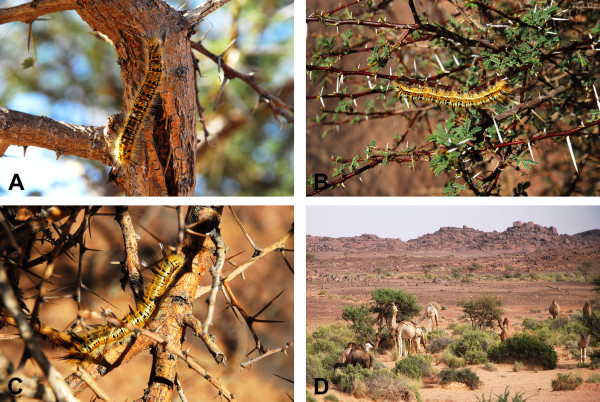
***Shedbera *on *Acacia tortilis* (A,B,C -Davide Rossi) and camels browsing *A. tortilis *leaves (D -Pavlína Kourková).** Pictures were taken during the field project implemented in November 2007 (**D**), December 2008 (**B**) and October 2009 (**A, C**) in the Zemmur region, Western Sahara.

The caterpillar that causes *duda* is a yellow and black caterpillar known by the Sahrawi as *shedbera* (Figure 3A–C). It is described as a hairy caterpillar feeding on *Acacia* species (Fabaceae), particularly on *Acacia tortilis* (Forssk.) Hayne – called *talha*, secondarily on *Acacia ehrenbergiana* Hayne – called *tamat* – and *Neurada procumbens* L. (Rosaceae) – called *saadan*. *Shedbera* is reported as present only ‘*when talha is green*’, and to have episodes of outbreaks in favourable conditions and usually after heavy rainfall. As Western Sahara camels feed largely from *Acacia tortilis* (Figure 3D) especially in periods of the year when *Acacia* leaves are amongst the only green pasture available (i.e. during springs and autumns and at the very beginning of rains), episodes of *duda* syndrome are described as the result of the interaction of *shedbera* outbreaks with the patterns of camel pasturing in Western Sahara.

Unfortunately, it was not possible to identify *shedbera* to the species level in absence of the adult moth. Its identity is discussed below, reducing it to few possible species in the genera *Lasiocampa*, *Psilogaster*, and *Streblote*, all belonging to the Lasiocampidae family. The family Lasiocampidae includes eggar moths, snout moths, lappet moths, and tent caterpillars. Larvae in this family are generally large, colourful, longitudinally striped, and densely hairy. They feed on the foliage of trees, and some species build communal webs (or ‘tents’) for protection from predators (like the caterpillars, the tents are also urticant as they are full of caterpillars’ hairs and setae). Females lay a large number of eggs, which in the right environmental conditions and abundance of foliage where to feed can lead to caterpillar outbreaks. *Lasiocampidae* caterpillars are known to cause itching, rashes, and eruption to people coming into contact with them [35]. The setae of these caterpillars are hollow cuticular tubes which pierce the skin and possibly inject toxic polypeptides and/or histamine, or other toxic agents. Unfortunately, little information about Lasiocampidae moths of the Sahara desert is available, except for their taxonomic classification.

Candidates for *shedbera* are *Lasiocampa trifolii* L. var. *mauritanica*, *Psilogaster loti algeriensis* Baker, and *Streblote acaciae* Klug. *Lasiocampa trifolii* is a Palearctic species living in Asia, Europe, and North Africa, and using Fabaceae species as host plants. In North Africa, it is present in Tunisia, Morocco (where it is sometimes named as subspecies *maroccana*), and Mauritania (*Lasiocampa trifolii* L. *mauritanica* or *Lasiocampa mauritanica* Staudinger), but no other information is present in the literature about its presence in the Sahara. *Psilogaster loti algeriensis* is distributed from Morocco to Libya; its larvae, however, commonly feed on *Cistus* species (Cistaceae) rather than on *Acacia* species. *Streblote acaciae* is distributed across North Africa, its caterpillars feed on *Acacia* species, and the adult flies from February until May [36].

According to Sahrawi, Moors, and Tuareg of Niger, *shedbera* caterpillars feed on *Acacia* leaves and have *Acacia* as primary host, leading to rare outbreaks during periods of *Acacia* foliaging. At the same time, camels pasturing in these areas feed largely from *Acacia*, which constitute a large share of all tree populations. Trees of *Acacia tortilis* are by far the most common trees in the Western Sahara/North Mauritanian areas and in the south of Tassili and Tibesti, areas where this species is a key forage for camels. The Sahrawi stated that caterpillar outbreaks on *Acacia* trees usually occur when they green after heavy rains (i.e. in autumn), but this is not always the case. In fact, they may be abundant even in the absence of rains (e.g. in spring), as ‘*talha becomes green at times even without rains*, *and it gets full of shedbera*.’ Thus, the cultural distribution of the *duda* syndrome seems to be the result of the overlap between areas of camel husbandry where *Acacia* trees represent a large proportion of the tree population, and areas where climatic and environmental conditions support periodical (albeit rare) outbreaks of *Lasiocampidae* caterpillars.

Do caterpillar outbreaks occur in the Sahara desert? The limited information available point to a positive answer although no outbreak of *Lasiocampidae* caterpillars has ever been reported in the Sahara. Nonetheless, caterpillars are dry-season pests in the central and western Sahelian ecosystem, as exemplified by the outbreaks and invasions of agricultural fields by *Spodopteru exemptu* Walk. (Noctuidae) – the African armyworm – in Ethiopia, Kenya, Somalia, Tanzania and Burundi in 1984 and 1999 [37], and by *Achaea catocaloides* Guenée (Noctuidae) in Liberia in 2009 [38]. Caterpillars of these species appear sporadically and suddenly in large outbreaks in Sahelian regions [39]. In the Sahara ecosystem, it is not rare to witness outbreaks of caterpillars of the striped hawk moth (*Hyles livornica* Esper, Sphingidae) during winter and spring after heavy rains [40]. One reason for the sudden mass appearances of *Hyles livornica* caterpillars in the desert environment is that the pupae might remain in diapause for more than a year, until stimulated to complete their metamorphosis by heavy rainfall. The emergence of adults from diapause at the same time helps to synchronize the population, as it facilitates mating, and eggs that are laid following those mating then become caterpillars that are able to take advantage of the annual plant growth that follows heavy rain [40].

A range of species belonging to the Lasiocampidae family that feed on *Acacia* tress and that may be responsible for the *duda* syndrome has been identified. Furthermore, it has been shown that caterpillar outbreaks are not unknown in the Sahara desert, although no outbreaks of species of the Lasiocampidae have been reported in the literature. The following section focuses on the caterpillar hypothesis by taking a bird’s-eye view of caterpillar-herbivore interactions and of reports of *duda*-like syndromes in other herbivores sourced from the literature.

### Caterpillar-borne reproductive loss syndromes in other herbivores

Is it possible that the ingestion by pregnant camels of Lasiocampidae caterpillars causes abortions and neonatal deaths as stated by Sahrawi, Moors, and Tuareg herders? Little information was found in the literature and in camel health manuals about such a possibility [41], and a search in Google Scholar (http://scholar.google.com/) and PubMed (http://www.ncbi.nlm.nih.gov/pubmed) of combinations of words such as ‘camel; caterpillar’ produced no relevant hits. Hence, the search was widened to veterinary entomology and environmental medicine related to cows, buffaloes, horses, and small ruminants, in an effort to proceed through analogical reasoning.

During spring 2001 in Kentucky, 532 early and late term abortions were reported in horses, corresponding to one third of the in-uterus foals [42]. On a smaller scale, the same happened in spring 2001 in south-eastern Ohio [43], and again in Kentucky in 2002 [44]. The syndrome, called Mare Reproductive Loss Syndrome (MRLS), was characterized by late-term foal losses, early-term foetal losses and weak foals [45], and by mild or no clinical signs in mares (about 15% of mares with MRLS exhibited, a few days before the outbreak of foetal loss, mild colitis and low fever, and others developed unilateral uveitis and non-specific bacterial infections in the aborted foetuses) [46]. Foals delivered live were weak, dehydrated, hypothermic, and dyspnoeic, evidenced bacterial infections and signs consistent with sepsis and disseminated inflammatory processes; placentas were oedematous and inflamed [47]. Weak foals survived for a short time (not more than four days) and eventually died of respiratory distress and cardiovascular collapse; some foals presented bilateral hyphema at birth [43,46,48]. During the abortion storm, high populations of Eastern Tent Caterpillars (ETC, *Malacosoma americanum* Fabricius, Lasiocampidae) wandering in pastures were observed, and an association between the two phenomena was soon recognized, suggesting that mares ingested the caterpillars while grazing, and that this ingestion caused widespread abortions. Mares that were less than 30 days pregnant during the period of exposure were apparently not affected, whereas mares at later stages of pregnancy had comparable exposure risk [49]. Following these events, an experimental study conducted in pigs fed using swine feed mixed with ETCs has shown that two out of five pigs aborted their entire litter in comparison with five control individuals [50]. In further studies, bacterial infections of the foetus/foetal membranes were supposed to cause the abortions, being sourced by intestinal penetration and diffusion into mares of septic barbed setal fragments, with significant pathological condition resulting only in those tissues that are poorly immunologically protected [42]. Setal fragments embed into the lining of the alimentary tract, penetrate the intestinal wall and enter into small blood vessels, and this facilitates the ingress of gut microbiota into the blood stream and their reproduction in sites with reduced immunity, such as the placenta and foetus [51]. Abortions are thus likely to be caused by microbial release from the mares’ gut and replication in the foetal fluids. Indeed, species of *Streptococcus* and *Actinobacillus* normally present in mares’ guts and oral cavities have been isolated in caterpillar-induced abortions, but not in caterpillars [52]. The septic penetrating setal emboli hypothesis is nowadays the most accepted (although still under scrutiny) in explaining MRLS: it is defined as ‘biologically unique and without precedent in the biological and medical literature’ [42]. In experimental conditions, Sebastian et al. [53] have shown that when the dose of and/or exposure to the caterpillars is large, pregnant mares are likely to abort rapidly (1–7 days); in contrast, when the exposure is shorter, the time to peak abortions is delayed, and abortions can occur after the caterpillar outbreak has ended, thus making their role in the syndrome difficult to identify. Since all penetration and distribution events are statistically determined, then the number of setal fragments entering the body (the number of caterpillars ingested) must be ‘optimally large’, and this explains why the effects are evident only with caterpillar outbreaks [51].

MRLS-like syndromes have also been reported recently in Australia. During 2004–05, an equine abortion storm similar to MRLS occurred in New South Wales following pregnant mares’ exposure to processionary caterpillars (*Ochrogaster lunifer* Herrich-Schaffer, Thaumetopoeidae), and/or their exoskeleton [54-56]. Although other hairy caterpillars were present, processionary caterpillars were the most abundant in the Australian region where the event has occurred [55]. The term Equine Amnionitis and Foetal Loss (EAFL) was established to describe the condition [57]. Other hypotheses related to the role of exposure of mares to the caterpillars of the White Cedar moth (*Leptocneria reducta* Walker, Lymantriidae) and of the Mistletoe Brown Tail Moth (*Euproctis edwardsii* Newman, Lymantriidae) are currently under investigation [57].

Another caterpillar-borne syndrome has been recently reported by Ethiopian pastoralists in the Bale Mountains: an urticating caterpillar associated with older and flowering *Erica* shrubs is recognized to cause ‘a skin rash on the cows and they become ‘dry’ and the milk production declines…it may eventually lead to their death’ [58]. The caterpillar was later identified as belonging to one of the families *Lasiocampidae*, *Notodontidae*, or *Lymantriidae*[58].

## Conclusions

In this paper, a camel reproductive loss syndrome observed by Sahrawi pastoralists of Western Sahara has been reported. This syndrome, called ‘*duda* syndrome’, is allegedly caused by the ingestion by pregnant camels of caterpillars that might appear in great numbers in *Acacia* treetops after heavy rainfall. Its manifestations are sudden abortions and the birth of weaklings, while the syndrome has no effect on the adult animals. Inductive and analogical reasoning, based on available circumstantiated evidence, was used to put forward a hypothesis to explain Sahrawi observations.

This hypothesis posits that the *duda* syndrome is caused by the ingestion by pregnant camels of caterpillars of the Lasiocampidae family (genera *Lasiocampa*, *Psilogaster*, and/or *Streblote*), and that, similarly to the aetiology described for the MRLS in the United States and the EAFL in Australia, and reproduced in experimental settings, caterpillars’ setal hairs act as septic penetrating emboli that cause bacterial infections and septicaemia to camel foetuses. If this hypothesis is true, it might imply that caterpillar-borne reproductive loss syndromes are more widespread (albeit probably characterised by rare onsets and depending on climatic and ecological happenstance) than was previously known, both in terms of the occurrence and of the number of species possibly involved (i.e. both wild/domestic herbivores and other caterpillar species). This would reveal a pattern of biological competition between caterpillars and herbivores, unknown until around a decade ago, and reported here for camels for the first time. Tobin et al. [51] foresaw that this competition pattern might be more widespread than is currently proven and, when discussing the septic penetrating setal emboli hypothesis, the authors stated: ‘If this setal hypothesis is correct, then similar exposure to mechanical and bacteriologically equivalent setae from other caterpillar species or from any other mechanically equivalent structure may also have the potential to produce syndromes akin to MRLS.’

Only further research will be able to provide clues if this hypothesis is true or should be rejected. First, the exact species of Lepidoptera allegedly involved in the abortion storms should be identified via adult specimens of moth. Once the species have been established, their caterpillars’ hairs should be examined under the microscope for setal barbs (which drive tissue penetration), and experimental studies (e.g. feeding the caterpillars to laboratory rats and looking for encapsulated setal fragments in the intestinal tract) should be conducted to test the hypothesis of the aetiological link with camel abortions. However, both logistical and financial constraints make a full field investigation of nomadic herds difficult, and these constraints are present in relation to the *duda* syndrome: epidemiological studies on camel populations scattered over a contested terrain are difficult to organize and perform, and camels are considered as marginal productive animals within the western-funding scientific world and thus their investigation is allocated less resource in comparison with other livestock species. This case study stands for the importance of investigating folk livestock diseases for their being based on knowledge and experience accumulated over centuries, and because they can reveal previously unknown biological phenomena that would deserve further investigation.

## Competing interests

The authors declare that they have no competing interests.

## Authors’ contributions

GV, DR, SMLS, and AB carried out field work. GV and ADN composed the literature review and drafted the manuscript. All authors read and approved the final manuscript. The view and findings in this article are solely those of the authors and do not necessarily reflect the views of the European Food Safety Authority.

## References

[B1] Vandebroek I Reyes-García V Albuquerque UP Bussmann R Pieroni A Local knowledge: Who cares? J Ethnobiol Ethnomed 2011 7 10.1186/1746-4269-7-35 PMC3286427 22113005

[B2] Mathias E Ethnoveterinary medicine in the era of evidence-based medicine: Mumbo-jumbo, or a valuable resource? Vet J 2007 173 241 242 16464626 10.1016/j.tvjl.2005.12.005

[B3] McCorkle CM, Mathias E, Schillhorn van Veen TW Ethnoveterinary Research and Development 1996 Intermediate Technology Publications, London

[B4] McCorkle CM An introduction to ethnoveterinary research and development J Ethnobiol 1986 6 129 149

[B5] Davis DK Quraishi K Sherman D Sollod A Stem C Ethnoveterinary medicine in Afghanistan: an overview of indigenous animal health care among Pashtun Koochi nomads J Arid Environ 1995 31 483 500

[B6] Raziq A Verdier de K Younas M Ethnoveterinary treatments by dromedary camel herders in the Suleiman mountainous region in Pakistan: an observation and questionnaire study J Ethnobiol Ethnomed 2010 6 10.1186/1746-4269-6-16 PMC3224957 20565919

[B7] Shen S Qian J Ren J Ethnoveterinary plant remedies used by Nu people in NW Yunnan of China J Ethnobiol Ethnomed 2010 6 10.1186/1746-4269-6-24 PMC2936877 20796273

[B8] Antoine-Moussiaux A Faye B Vias G Tuareg ethnoveterinary treatments of camel diseases in Agadez area (Niger) Trop Anim Health Prod 2007 39 83 89 18318345 10.1007/s11250-007-4404-1

[B9] Bekele T Studies on the respiratory disease ‘sonbobe’ in camels in the eastern lowlands of Ethiopia Trop Anim Health Prod 1999 31 333 345 10599129 10.1023/a:1005290523034

[B10] Dirie MF Abdurahman O Observations on little known diseases of camels (Camelus dromedarius) in the Horn of Africa Rev Sci Tech Off Int Epiz 2003 22 1043 1049 10.20506/rst.22.3.1456 15005561

[B11] Caratini S Les Rgaybat (1610–1934). 1: Des chameliers à la conquete d'un territoire 1989 L'Harmattan, Paris

[B12] Caratini S Les Rgaybat (1610–1934). 2: Territoire et société 1989 L'Harmattan, Paris

[B13] Caro Baroja J Estudios saharianos 1955 Instituto de Estudios Africanos, Madrid

[B14] Loewenberg S Displacement is permanent for the Sahrawi refugees Lancet 2005 365 1295 1296 15828081 10.1016/S0140-6736(05)61010-0

[B15] San Martin P Western Sahara: The Refugee Nation 2010 University of Wales Press, Cardiff

[B16] Bathia M The Western Sahara under Polisario control Review of African Political Economy 2001 28 291 301

[B17] Broglia A Volpato G Pastoralism and displacement: strategies and limitations in livestock raising by Sahrawi refugees after thirty years of exile Journal of Agriculture and Environment for International Development 2008 102 105 122

[B18] Brooks N Chiapello I Di Lernia S Drake N Legrand M Moulin C Prospero J The climate-environment-society nexus in the Sahara from prehistoric times to the present day The Journal of North African Studies 2005 10 253 292

[B19] Soler N Serra C Escola J Unge J Sahara Occidental: Pasado y Presente de un Pueblo 1999 Universidad de Girona, Girona

[B20] Bernard HR Handbook of Methods in Cultural Anthropology 1998 Altamira Press, Walnut Creek, CA, USA

[B21] Code of ethics of the American Anthropological Association http://www.aaanet.org/issues/policy-advocacy/Code-of-Ethics.cfm

[B22] International Society of Ethnobiology ISE Code of Ethics (with 2008 additions) 2006 http://ethnobiology.net/code-of-ethics/

[B23] Flint Taylor R Njaa BL Njaa BL General Approach to Fetal and Neonatal Loss Kirkbride's Diagnosis of Abortion and Neonatal Loss in Animals 2012 4 Wiley- Blackwell 1 12

[B24] Wernery U Kaaden OR Infectious Diseases of Camelids 1995 Blackwell Wissenschafts-Verlag, Berlin

[B25] Ahmed SM Hegde BP Gahlot TK Preliminary study on the major important camel calf diseases and other factors causing calf mortality in the Somali Regional state of Ethiopia Recent trends in camelids research and future strategies for saving camels 2007 Rajasthan, India 31 41

[B26] Gutierrez C Corbera JA Juste MC Doreste F Morales I An outbreak of abortions and high neonatal mortality associated with Trypanosoma evansi infection in dromedary camels in the Canary Islands Vet Parasitol 2005 130 163 168 15893083 10.1016/j.vetpar.2005.02.009

[B27] Dioli M Stimmelmayr R Schwartz HJ, Dioli M Important camel diseases The one-humped camel (C dromedarius) in Eastern Africa 1992 Verlag Josef Margraf, Weikersheim 155 224

[B28] Tibary A Anouassi A Skidmore L, Adams GP Neonatal care in camelids Recent Advances in Camelid Reproduction 2001 International Veterinary Information Service, Ithaca, NY

[B29] Dioli M Pictorial Guide to Traditional Management 2007 Husbandry and Diseases of the One-Humped Camel, Photographic CD-ROM

[B30] Antoine-Moussiaux A Faye B Vias G Connaissances ethnovétérinaires des pathologies camélines dominantes chez les Touaregs de la région d’Agadez (Niger) 2006 1 21 http://camelides.cirad.fr/fr/science/index.html

[B31] El Hadi Ould Taleb M Generalites sur l'elevage du dromadaire en Mauritanie 1998 FAO, Rome 10

[B32] Kane Y Diop C Isselmou E Kaboret Y Ould Mekhalle M Diallo BC Contraintes majeures de l'élevage camelin en Mauritanie Revue Africaine de Santé et de Productions Animales 2003 1 31 37

[B33] Dia ML Diop A Ahmed OM Diop C El Hacen OT Diarrhées du chamelon en Mauritanie: résultats d'enquête Rev Elev Med Vet Pays Trop 2000 53 149 152

[B34] Diagana D Contribution à l’étude de l’élevage du dromadaire en Mauritanie Ecole Inter Etats Sci. Méd. vét 1977

[B35] Hellier FF Warin RP Caterpillar dermatitis Br Med J 1967 2 346 348 6023131 10.1136/bmj.2.5548.346 PMC1841743

[B36] Rougeot PC Viette P Guide des papillons nocturnes d'Europe et d'Afrique du Nord 1978 Delachaux et Niestlé, Lausanne

[B37] Rose DJW Dewhurst CF Page WW The African Armyworm Handbook: The Status, Biology, Ecology, Epidemiology and Management of Spodoptera exempta (Lepidoptera: Noctuidae) 2000 Natural Resources Institute, Greenwich

[B38] Liberia identifies caterpillar species eating crops http://uk.reuters.com/article/idUKL3475648._CH_.2420

[B39] Pedgley DE Page WW Mushi A Odiyo P Amisi J Dewhurst CF Dunstan WR Fishpool LDC Harvey AW Megenasa T Rose DJW Onset and spread of an African armyworm upsurge Ecological Entomology 1989 14 311 333

[B40] Hornby D Hawk moth caterpillars ENHG focus 2007 2007 3 4

[B41] Bizimana N Traditional Veterinary Practice in Africa 1994 GTZ, Eschborn

[B42] The 2001 Kentucky Equine Abortion Storm The Caterpillar/Setal Hypothesis of the Mare Reproductive Loss Syndrome (MRLS) http://thomastobin.com/mrlstox.htm

[B43] Frazer GS Powell DG, Troppman A, Tobin T Mare Reproductive Loss Syndrome in Southeastern Ohio, Spring 2001 First Workshop on Mare Reproductive Loss Syndrome, 2002 2003 The College of Agriculture, University of Kentucky 36 37

[B44] Powell DG Troppman A Tobin T Proceedings of the First Workshop on Mare Reproductive Loss Syndrome 2003 The College of Agriculture, University of Kentucky, Lexington, Kentucky

[B45] Schlafer DH Powell DG, Troppman A, Tobin T Placental Toxicology: Recognized Placental Toxicants in Veterinary Medicine and Consideration of a Likely Role of a Placental Toxicant in Mare Reproductive Loss Syndrome First Workshop on Mare Reproductive Loss Syndrome, 2002 2003 The College of Agriculture, University of Kentucky 44 47

[B46] Byars TD Seahorn TL Powell DG, Troppman A, Tobin T Clinical Observations of Mare Reproductive Loss Syndrome in Critical Care Mares and Foals First Workshop on Mare Reproductive Loss Syndrome, 2002 2003 The College of Agriculture, University of Kentucky 15 16

[B47] Brown S Powell DG, Troppman A, Tobin T Field and Clinical Observations Related to Late Fetal Loss in Mares Affected with Mare Reproductive Loss Syndrome First Workshop on Mare Reproductive Loss Syndrome, 2002 2003 The College of Agriculture, University of Kentucky 14 15

[B48] Sebastian MM Bernard WV Riddle TW Latimer CR Fitzgerald TD Harrison LR Review Paper: Mare Reproductive Loss Syndrome Vet Pathol 2008 45 710 722 18725479 10.1354/vp.45-5-710

[B49] Riddle TW Powell DG, Troppman A, Tobin T Clinical Observations Associated with Early Fetal Loss in Mare Reproductive Loss Syndrome during the 2001 and 2002 Breeding Seasons First Workshop on Mare Reproductive Loss Syndrome, 2002 2003 The College of Agriculture, University of Kentucky 12 14

[B50] O'Rourke K Eastern tent caterpillars implicated in swine abortions J Am Vet Med Assoc 2003 223 1406

[B51] Tobin T Harkins JD Roberts JF VanMeter PW Fuller TA The Mare Reproductive Loss Syndrome and the Eastern Tent Caterpillar II: A Toxicokinetic/Clinical Evaluation and a Proposed Pathogenesis: Septic Penetrating Setae International Journal of Applied Research in Veterinary Medicine 2004 2 142 158

[B52] Donahue JM Sells SF Bolin DC Classification of Actinobacillus spp isolates from horses involved in mare reproductive loss syndrome Am J Vet Res 2006 67 1426 1432 16881857 10.2460/ajvr.67.8.1426

[B53] Sebastian MM Gantz MG Tobin T Harkins JD Bosken JM Hughes C Harrison LR Bernard WV Richter DL Fitzgerald TD The mare reproductive syndrome and the eastern tent caterpillars: a toxicokinetic/statistical analysis with clinical, epidemiologic, and mechanistic implications Veterinary Therapy 2003 4 324 339 15136975

[B54] Perkins NR Sebastian MM Todhunter KH Wylie RM Begg AP Gilkerson JR Racklyeft DJ Chicken C Wilson MC Cawdell-Smith AJ Panter KE, Wierenga TL, Pfister JA Pregnancy loss in mares associated with exposure to caterpillars in Kentucky and Australia Poisonous Plants: Global Research and Solutions 2007 CABI Publishing, Wallingford, Oxford 165 169

[B55] Cawdell-Smith AJ Todhunter KH Perkins NR Bryden WL Processionary caterpillars are an abortifacient in mares Proceedings of the Australian Society for Animal Production 2008 27 75

[B56] Cawdell-Smith AJ Todhunter KH Anderson ST Perkins NR Bryden WL Equine amnionitis and fetal loss: Mare abortion following experimental exposure to Processionary caterpillars (Ochrogaster lunifer) Equine Vet J 2012 44 282 288 21815917 10.1111/j.2042-3306.2011.00424.x

[B57] Todhunter KH Perkins NR Wylie RM Chicken C Blishen AJ Racklyeft DJ Muscatello G Wilson MC Adams PL Gilkerson JR Equine amnionitis and fetal loss: The case definition for an unrecognised cause of abortion in mares Aust Vet J 2009 87 35 38 19178475 10.1111/j.1751-0813.2008.00386.x

[B58] Johansson MU Fetene M Malmer A Granström A Tending for cattle: traditional fire management in Ethiopian montane heathlands Ecol Soc 2012 17 19

